# Effects of a water-soluble formulation of tylvalosin on disease caused by porcine reproductive and respiratory syndrome virus alone in sows or in combination with *Mycoplasma hyopneumoniae* in piglets

**DOI:** 10.1186/s12917-023-03571-x

**Published:** 2023-02-01

**Authors:** Alfonso Lopez Rodriguez, Veronica L. Fowler, Michael Huether, David Reddick, Christine Tait-Burkard, Marie O’Shea, Stephanie Perkins, Nirosh Dias, Robin Buterbaugh, Hafid A. Benchaoui

**Affiliations:** 1 ECO Animal Health Ltd, London, UK; 2 Moredun Scientific Ltd, Pentlands Science Park, Bush Loan, Penicuik, Midlothian, EH26 0PZ UK; 3 The Roslin Institute and Royal (Dick) School of Veterinary Studies, University of Edinburgh, Easter Bush, Midlothian, EH25 9RG UK; 4 RTI, LLC, 801 32nd Ave, Brookings, SD 57006 USA

**Keywords:** Tylvalosin, Macrolide, *Mycoplasma hyopneumoniae*, Porcine reproductive and respiratory virus, PRRS, Immunomodulation, Pro-inflammatory cytokines

## Abstract

**Background:**

The effect of a water-soluble formulation of tylvalosin (Aivlosin® 625 mg/g granules) on disease caused by porcine reproductive and respiratory syndrome virus (PRRSV) and *Mycoplasma hyopneumoniae* (*Mhyop*) was investigated in two animal studies*.* In a PRRSV challenge model in pregnant sows (*n* = 18), six sows received water medicated at target dose of 5 mg tylvalosin/kg body weight/day from 3 days prior to challenge until the end of gestation. Six sows were left untreated, with a third group remaining untreated and unchallenged. Sows were challenged with PRRSV-2 at approximately 85 days of gestation. Cytokines, viremia, viral shedding, sow reproductive parameters and piglet performance to weaning were evaluated. In a dual infection study (*n* = 16), piglets were challenged with *Mhyop* on days 0, 1 and 2, and with PRRSV-1 on day 14 and euthanized on day 24. From day 10 to 20, eight piglets received water medicated at target dose of 20 mg tylvalosin/kg body weight/day and eight piglets were left untreated. Cytokines, viremia, bacteriology and lung lesions were evaluated.

**Results:**

In the PRRSV challenge study in pregnant sows, tylvalosin significantly reduced the levels of serum IL-8 (*P* < 0.001), IL-12 (*P* = 0.032), TNFα (*P* < 0.001) and GM-CSF (*P* = 0.001). IL-8 (*P* = 0.100) tended to be lower in uterus of tylvalosin sows. All piglets from tylvalosin sows surviving to weaning were PRRSV negative in faecal swabs at weaning compared to 33.3% PRRSV positive piglets from untreated sows (*P* = 0.08).

In the dual challenge study in piglet, tylvalosin reduced serum IL1β, IL-4, IL-6, IL-8, IL-10, IL-12, IL-1α, IL-13, IL-17A, IL-18, GM-CSF, TGFβ1, TNFα, CCL3L1, MIG, PEPCAM-1 (*P* < 0.001) and increased serum IFNα, IL-1ra and MIP-1b (*P* < 0.001). In the lungs, tylvalosin reduced IL-8, IL-10 and IL-12 compared to untreated pigs (*P* < 0.001) and tended to reduce TNFα (*P* = 0.082). Lung lavage samples from all tylvalosin treated piglets were negative for *Mhyop* (0 cfu/mL) compared to the untreated piglets which had mean *Mhyop* counts of 2.68 × 10^4^ cfu/mL (*P =* 0.023).

**Conclusion:**

Overall, tylvalosin reduced both local and systemic proinflammatory cytokines after challenge with respiratory pathogens in sows and in piglets. Tylvalosin was effective in reducing *Mhyop* recovery from the lungs and may reduce virus shedding in piglets following transplacental PRRSV infection in sows.

## Background

Porcine respiratory disease is a complex condition, often involving more than one pathogen. *Mycoplasma hyopneumoniae* (*Mhyop*), the primary pathogen of enzootic pneumonia in pigs, occurs worldwide and causes major economic losses to the pig industry [1]. The organism adheres to and damages the ciliated epithelium of the respiratory tract [1, 2]. Affected pigs show chronic coughing, are more susceptible to other respiratory infections and have a reduced performance in growth rate [3].

Porcine reproductive and respiratory syndrome (PRRS) virus (PRRSV) is globally endemic to pig herds and results in considerable economic losses in the sector as well as considerable welfare issues for individual animals on infected properties [4–6]. In piglets, PRRSV can result in loss of condition, inappetence, an acute and extensive pneumonia (coughing, sneezing, increased respiratory rate and pyrexia), diarrhoea and, in some cases, lameness [6]. It also predisposes pigs to *Streptococcus suis* (*S. suis*) septicaemia [6] . In breeding sows, PRRSV induces reproductive failures with increased stillbirths, abortions, and mummifications of piglets as well as increased mortality in piglets that are born alive [6].

Both pathogens are components of the Porcine Respiratory Disease Complex (PRDC) and in many cases, respiratory disease in pigs is much more severe because of a combination of different pathogens rather than a single pathogenic agent [2, 5, 7]. It has also been shown that respiratory disease in pigs is exacerbated by intensive inflammation mediated by pro-inflammatory cytokines [7, 8].

During infection, PRRSV can induce alterations of immunoregulatory cytokines [7, 8]. These cytokines are known amongst other activities to stimulate chemotaxis and degranulation of neutrophils but may, under some circumstances, result in an uncontrolled immune response which can have deleterious effects on lung tissue and exacerbate the severity of the clinical presentation, described previously as a “cytokine storm” [9, 10].

Cases of respiratory disease are often treated with antimicrobials, and it has been suggested that macrolides have the added benefit of anti-inflammatory properties in addition to their antimicrobial activity [11–13]. A potential antiviral effect has also been demonstrated in vitro for some macrolides although results in vivo are inconclusive [14, 15].

Tylvalosin (3–0-acetyl-4″-0-isovaleryltylosin) is a 16-membered ring macrolide antibiotic developed solely for use in veterinary medicine [16]. When administered orally, tylvalosin is effective in the treatment of several porcine pathogens, including *Mhyop*, *Lawsonia intracellularis* and *Brachyspira hyodysenteriae*. Efficacy of tylvalosin against *Mhyop* is well described [17]. In in vitro studies, tylvalosin has been shown to induce apoptosis of porcine neutrophils and in macrophages, promote efferocytosis, inhibit cytokines such as IL-6, IL-8, IL-1α, TNFα and leukotriene B_4_ (LTB_4_) production and to induce and release pro-resolving Lipoxin A4 and Resolvin D1 [18, 19]. In addition, activity against PRRSV has been described in vitro for tylvalosin, but in vivo data are scarce [14, 20].

The potential for tylvalosin to modulate the immune response and its spectrum to ameliorate clinical signs therefore needs further investigation. The objective of these studies was to further elucidate the immunomodulatory activity of tylvalosin and whether there are any measurable anti-viral effects in vivo, through the administration of tylvalosin to sows prior to challenge with PRRSV, or to piglets sequentially challenged with *Mhyop* and PRRSV.

## Results

### Single challenge sow study

#### Clinical observations in sows

**Fig. 1:**
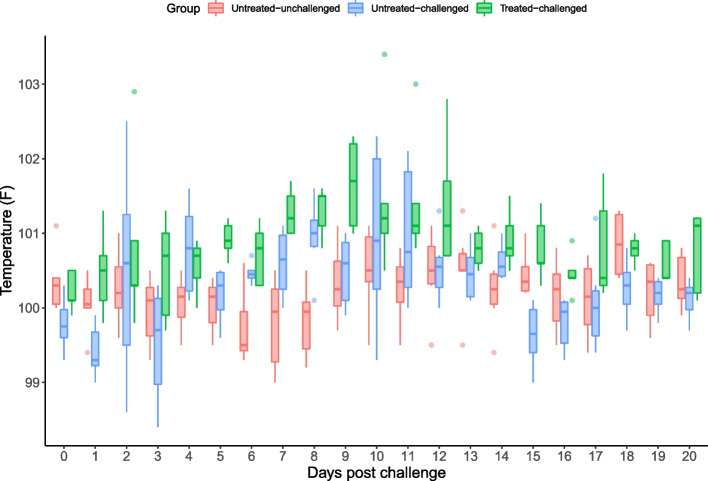
Distribution of sow’s daily temperatures per group in the PRRSV challenge study in pregnant sows. The box-and-whisker plots depict the minimum, first quartile, median, third quartile and the maximum temperature values observed at each day within each group. Individual points are observed outliers. Treatment description is in Table 7

All sows from the untreated-challenged (T02) and treated-challenged (T03) groups increased rectal temperatures from baseline, whereas the untreated-unchallenged sows (T01) did not (Fig. 1). Untreated-challenged sows had significantly higher rectal temperatures (*P* = 0.012) when compared to untreated-unchallenged sows for the time period of 5–10 days post challenge. Rectal temperatures were significantly higher in tylvalosin treated-challenged sows on occasional days when compared to those from group T01 and T02 (*P* < 0.001), although temperatures remained within normal physiological range in all groups. There were no significant differences between groups in any of the other clinical observations (demeanour, nasal discharge, coughing and respiratory rate) (data not shown). One tylvalosin sow was euthanised 17 days post challenge due to inappetence. At *post-mortem*, this sow was observed to have a large gastric ulcer. One untreated-unchallenged sow was found dead two days post farrow. At *post-mortem*, this sow was observed to have died of sepsis diagnosed from a *Streptococcus suis* and *Pasteurella multocida* positive lung culture.

#### Cytokine response in sow’s serum

**Fig. 2:**
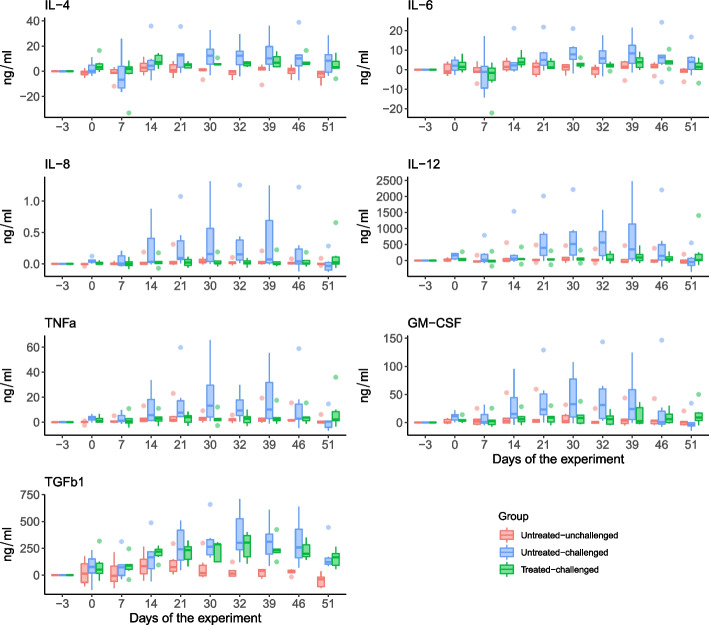
Changes in cytokine production in time from the start of the experiment in PRRSV challenge study in pregnant sows. The box-and-whisker plots depict the minimum, first quartile, median, third quartile and the maximum temperature values observed at each day within each group. Individual points are observed outliers. Statistical differences are described in the text of the manuscript. Treatment description is in Table 7

Untreated-challenged sows had significantly higher IL-4 and IL-6 when compared to untreated-unchallenged sows (*P* = 0.03 and *P* = 0.021; Fig. 2) whereas tylvalosin treated sows were not different from untreated-unchallenged sows (*P* = 0.08 and *P* = 0.253; Fig. 2). IL-8 was significantly higher from untreated-challenged sows when compared to untreated-unchallenged sows (*P* < 0.001) and tylvalosin treated-challenged sows (*P* < 0.001; Fig. 2). IL-12 was significantly higher in untreated-challenged sows when compared to untreated-unchallenged (*P* = 0.018) and tylvalosin treated-challenged sows (*P* = 0.032; Fig. 2). TNFα was statistically higher in untreated-challenged sows when compared to tylvalosin treated-challenged sows (*P =* 0.004) but not statistically higher compared to untreated-unchallenged (*P* = 0.210) (Fig. 2). GM-CSF was higher in untreated-challenged sows when compared to untreated-unchallenged (*P* = 0.054) and to treated-challenged sows (*P* = 0.03) (Fig. 2). TGFβ1 was significantly higher in untreated-challenged sows (*P* = 0.014) and tylvalosin treated-challenged sows (*P* < 0.001) when compared to untreated-unchallenged sows (Fig. 2). IL-10 was numerically higher in untreated-challenged sows when compared to untreated-unchallenged sows (*P* = 0.204) and treated-challenged sows (*P* = 0.114), but this was not statistically significant (Fig. 2).

#### Cytokine response in sow’s uterus

Biomarkers measured in the fluid collected from lumen from the reproductive tract are summarised in Table 1. Twenty cytokines were also evaluated in sow reproductive tract fluid collected at post-mortem. Of the 20 cytokines screened, there was no significant differences between any of the groups, however there was one observation near to statistical significance. Untreated-unchallenged sows had lower TNFα when compared to untreated-challenged sows (Table 1; *P* = 0.076). Although not statistically different, compared to untreated sows, tylvalosin reduced expression of IL-8, IL-10, IL-12 and TNFα by nearly 50% keeping the levels similar to untreated-unchallenged sows (Table 1).

**Table 1:** Biomarkers measured in the fluid recovered from the reproductive tract of sows in the PRRSV challenge study in pregnant sows. Samples were collected after euthanasia approximately 21 days post farrow. Treatement description is in Table 5

Biomarker	T01 Untreated-unchallenged	T02 Untreated-challenged	T03 Treated-challenged	***p***.value T02vs T01	***p***.value T02vsT03	***p***.value T03vsT01
CCL3L1	252.46 ± 282.01	214.86 ± 189.28	687.57 ± 601.19	0.807	0.215	0.252
GM-CSF	405.76 ± 231.15	1636.01 ± 1865.2	496.69 ± 373.04	0.168	0.200	0.688
IFNa	65,035.47 ± 29,915.14	57,990.61 ± 20,895.11	48,621.73 ± 16,753.99	0.670	0.457	0.336
IFNg	2848.39 ± 3172.53	1404 ± 1616.26	858.35(296.91, 5390.5)	0.393	1.000	0.607
IL-10	275.33 ± 177.47	543.01 ± 501.47	256.43 ± 83.5	0.266	0.224	0.840
IL-12p40p70	16,785.44 ± 7702.19	46,381.72 ± 45,447.95	24,129.79 ± 13,604.8	0.174	0.302	0.384
IL-13	3073.07 ± 2044.33	2813.8 ± 1435.92	2128.84 ± 301.75	0.818	0.304	0.363
IL-17A	71.54 ± 56.64	62.18 ± 38.44	126.28 ± 127.6	0.763	0.394	0.470
IL-18	3,407,125.07 ± 2,641,856.22	2,343,833.69 ± 831,018.25	2,547,434.94 ± 675,746.05	0.429	0.683	0.518
IL-1a	84,130.91 ± 66,856.55	54,783.06 ± 29,747.06	70,931.41 ± 44,128.24	0.403	0.551	0.733
IL-1b	1318.08 ± 1140.23	1199.56 ± 512.52	1146.54 ± 380.62	0.838	0.856	0.765
IL-1ra	3694.59 ± 4147.84	6244.39 ± 8219.92	1845.21 ± 1088.76	0.526	0.250	0.386
IL-4	3701.24 ± 3038.7	4479.77 ± 1604.22	5782 ± 4597.23	0.625	0.619	0.471
IL-6	1448.03 ± 1142.23	1558.83 ± 627.73	1744.6 ± 928	0.853	0.741	0.680
IL-8	17.31 ± 10.37	41.92 ± 38.88	10.29 ± 7.55	0.189	0.105	0.279
MIG	3821.29 ± 2765.76	2720.92 ± 954.38	3077.41 ± 1720.89	0.436	0.724	0.637
MIP-1b	13.84 ± 10.81	12.51 ± 11.71	69.37 ± 85.48	0.849	0.276	0.285
PECAM-1	3926.3 ± 2141.81	4269.73 ± 2656.54	2853.67 ± 814.45	0.818	0.265	0.347
TGFb1	221,572.86 ± 114,642.43	244,924.97 ± 72,559.89	256,329.02 ± 92,330.07	0.706	0.843	0.630
TNFa	26,530.25 ± 12,185.13	82,296.32 ± 61,218.41	35,245.93 ± 17,273.66	0.076	0.125	0.431

#### Viral load in sows

There was no significant difference in PRRSV viral load in serum, oral and faecal swabs between untreated-challenged group and treated-challenged sows (Table 2). There were no significant differences between untreated- challenged sows and treated-challenged sows in viral load for any of the organ samples assessed at *post-mortem* and none of the sows in any group had lung lesions (*P >* 0.05). All sows in the untreated-unchallenged group remained PRRSV negative in all samples throughout the study.

**Table 2:** Mean PRRSv PCR treshold cycle (Ct) values for different samples collected from sows in the PRRSV challenge study in pregnant sows. Samples were collected on different days post challenge and days post-farrow. Values equal or higher than 37 were considered as not detected (nd) and are not included in the calculation of the mean Ct values. Values between brackets indicate the ratio of positive samples by total number of samples

		Mean Ct values of positive samples (number of positive samples/ number of samples)									
		Days post challenge					Days post-farrow				
Sample type	Group	-3	0	7	14	21	0	2	9	16	21–25
Faecal swab	Untreated-unchallenged	ns	nd (0/6)	nd (0/6)	nd (0/6)	nd (0/6)	nd (0/6)	nd (0/5)	nd (0/5)	nd (0/5)	nd (0/5)
	Untreated-challenged	ns	nd (0/6)	30.1 (4/6)	34.6 (4/6)	33.6 (3/6)	34.1 (4/6)	33.4 (1/6)	31.7 (1/6)	nd (0/6)	nd (0/6)
	Treated-challenged^a^	ns	nd (0/6)	30.6 (3/6)	33.3 (3/6)	32.4 (1/5)	33.5 (4/5)	31.6 (2/5)	32.4 (2/5)	nd (0/5)	nd (0/5)
Oral swab	Untreated-unchallenged	ns	nd (0/6)	nd (0/6)	nd (0/6)	nd (0/6)	nd (0/6)	nd (0/5)	nd (0/5)	nd (0/5)	nd (0/5)
	Untreated-challenged	ns	nd (0/6)	33.4 (4/6)	35.4 (1/6)	nd (0/6)	nd (0/6)	36.0 (1/6)	nd (0/6)	36.8 (1/6)	nd (0/6)
	Treated-challenged^a^	ns	nd 0/6)	32.9 (3/6)	nd (0/6)	nd (0/5)	35.7 (1/5)	33.8 (1/5)	nd (0/5)	nd (0/5)	nd (0/5)
Blood serum	Untreated-unchallenged	nd (0/6)	nd (0/6)	nd (0/6)	nd (0/6)	nd (0/6)	nd (0/6)	nd (0/6)	nd (0/5)	nd (0/5)	nd (0/5)
	Untreated-challenged	nd (0/5)	nd (0/6)	25.8 (6/6)	32.1 (6/6)	32.6 (6/6)	33.3 (5/6)	33.4 (4/6)	nd (0/6)	nd (0/6)	36.4 (2/6)
	Treated-challenged^a^	nd (0/6)	nd (0/6)	25.4 (6/6)	32.7 (6/6)	33.2 (4/5)	34.1 (4/5)	32.9 (3/5)	35.6 (2/5)	34.3 (2/5)	31.9. (1/5)

*ns* no sample

*nd* not detected (Ct ≥ 37)

^a^Sows treated with tylvalosin at 5 mg/kg per day in drinking water from 3 days before challenge until farrowing

#### Viral load and lung lesions in piglets

There was no significant difference in PRRSV viral load in piglets derived from either untreated-challenged sows or treated- challenged sows in serum, oral swabs or tissue samples (Table 3). However, none of the surviving piglets (0%) from treated-challenged sows had positive faecal swabs compared to three pigs (33.3%) from untreated sows (*P* = 0.08). None of the surviving piglets (0%) from treated-challenged sows had lung lesions at weaning compared to two pigs (22.2%) from untreated sows (*P* = 0.503). All piglets from untreated-unchallenged sows remained PRRSV negative in all samples throughout the study.

**Table 3:** Mean PRRSv PCR treshold cycle (Ct) values for different samples collected from farrowed piglets in the PRRSV challenge study in pregnant sows. Samples were collected on different days post challenge and days post-farrow. *V*alues equal or higher than 37 were considered as not detected (nd) and are not included in the calculation of the mean Ct values. Values between brackets indicate the ratio of positive samples by total number of samples

		Mean Ct values of positive samples (number of positive samples/ number of samples)				
		Days post-farrow				
		0	2	9	16	21–25
Faecal swab	Untreated-unchallenged	nd (0/95)	nd (0/70)	nd (0/64)	nd (0/63)	nd (0/61)
	Untreated-challenged	33.3 (14/41)	30.9 (29/29)	30.6 (10/10)	35.5 (7/8)	33.9 (3/8)
	Treated-challenged^a^	34.0 (19/36)	31.5 (26/28)	30.4 (12/13)	32.8 (5/5)	nd (0/9)
Oral swab	Untreated-unchallenged	nd (0/95)	nd (0/70)	nd (0/64)	nd (0/63)	nd (0/60)
	Untreated-challenged	35.2 (12/41)	32.5 (21/29)	34.9 (6/10)	33.2 (4/8)	34.5 (3/8)
	Treated-challenged^a^	34.2. (23/36)	34.4 (18/28)	33.1 (10/13)	34.6 (7/10)	35.8 (2/10)
Blood serum	Untreated-unchallenged	nd (0/93)	nd (0/69)	nd (0/64)	nd (0/62)	nd (0/60)
	Untreated-challenged	22.3 (36/41)	16.0 (28/28)	17.1 (10/10)	19.2 (7/7)	23.8 (8/8)
	Treated-challenged^a^	19.7. (34/35)	16.7 (28/28)	16.0 (13/13)	18.1 (10/11)	24.0 (9/9)

*ns* no sample

*nd* not detected (Ct ≥ 37)

^a^Sows treated with tylvalosin at 5 mg/kg per day in drinking water from 3 days before challenge until farrowing

#### Reproductive performance

The mean gestation length appeared lower in untreated-challenged sows (114.7 days) compared to tylvalosin treated-challenged sows (116.6 days) and to untreated-unchallenged sows (116.2 days), although this parameter was not compared statistically.

On average, the number of piglets born per sow was 16.3, 17.3 and 21.0 for the untreated-unchallenged (*n* = 6), the untreated-challenged (*n* = 6) and the treated-challenged group, respectively (*n* = 5). The mean number of piglets that were either stillborn, non-viable or mummies was 9.7 for untreated-challenged sows and 13.8 for treated-challenged sows, and both were higher than those born to untreated-unchallenged sows (0.5 piglets per sow). The number of piglets born alive to untreated-challenged sows (7.5 mean piglets per sow) and treated-challenged sows (7.2 mean piglets per sow), were both lower than those born to untreated-unchallenged sows (15.8 mean piglets per sow).

**Fig. 3:**
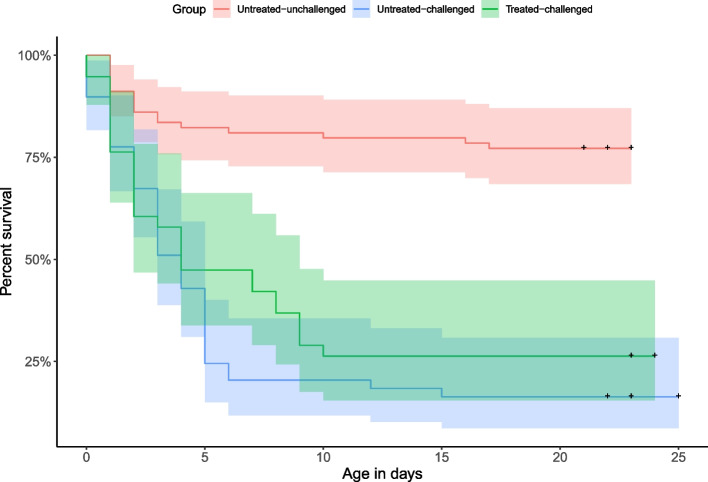
Group piglet survival curves in PRRSV challenge study in pregnant sows. Shaded areas are the corresponding 95% confidence intervals. Statistical differences are described in the text of the manuscript. Treatment description is in Table 7

**Fig. 4:**
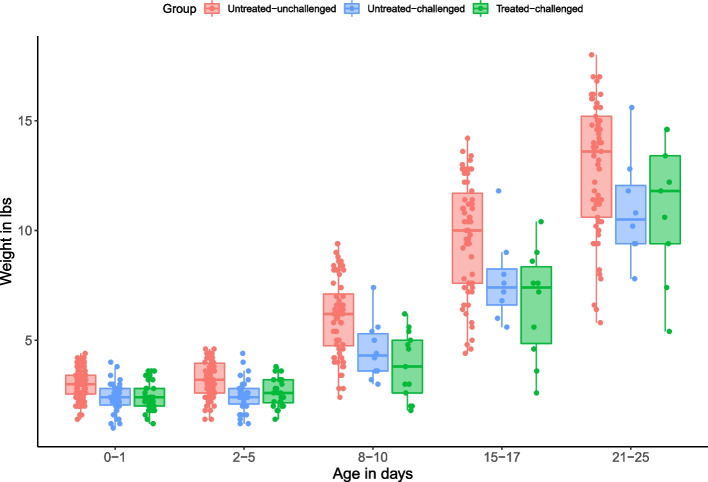
Distribution of body weights of surviving piglets during the course of the experiment. Statistical differences are described in the text of the manuscript. Treatment description is in Table 7

The probability of piglets surviving was significantly higher in piglets from untreated-unchallenged sows when compared to untreated-challenged sows and treated-challenged sows (*P* < 0.001) (Fig. 3). Median survival time [95% confidence intervals (CI)] for piglets from untreated-challenged sows was 4 (95%CI: 3–5) days and for piglets from tylvalosin treated-challenged sows was 4 (95CI: 2–9) days. The probability of piglet survival to weaning was not statistically different for the progeny from tylvalosin treated-challenged sows than for that from untreated-challenged sows (26% vs 16%; *P* = 0.486). The number of piglets surviving to weaning from treated-challenged sows was 2.8 piglets/sow compared to 1.5 piglets/sow from untreated-challenged sows. Piglet weight gain was significantly lower in piglets from both untreated-challenged and treated-challenged sows (*P* < 0.001) than that of piglets from untreated-unchallenged sows. The weights of piglets from treated-challenged sows was not different on average (11.04 Ibs) than those of untreated-challenged sows (10.97lbs) (Fig. 4).

### Piglet dual challenge study

#### Piglet performance and clinical disease

Weight gain and feed conversion in treated-challenged pigs was greater (+1Kg) than observed in the untreated-challenged piglets, however this was not statistically significant (Table 4). No statistically significant difference was noted in composite clinical scores between groups on evaluation days 14 and 24 (Table 4), or in lung congestion scores (Table 5), however there was a numerical reduction in lung consolidation scores of 34% in tylvalosin treated-challenged piglets compared to untreated-challenged piglets (Table 5).

**Table 4:** Piglet performance and clinical scores in the dual challenge study in piglets. All piglets were challenged with *M. hyopneumoniae* on days 0, 1 and 3 and with PRRSV on day 14. Pigs in treated-challenged group were medicated daily with tylvalosin at 20 mg/kg body weight from day 10 to day 20. Treatement description is in Table 5

Group	Number of pigs on day 0	Mortality count	Body weights (BW) mean ± SD in kg			Feed conversion ratio Day 14-Day 24^*^
			BW Day 0	BW Day 24	BW gain Day 0-DAy 24	
Treated- challenged	8	1 (day 22)^a^	9.39 ± 1.77	16.35 ± 3.19	7.1 ± 2.0	1.85
Untreated-challenged	8	1 (day 21)^b^	8.94 ± 1.37	14.83 ± 3.22	6.1 ± 2.7	2.62
*P*-value		Not compared	Not compared		*P* = 0.387	Not compared

*Calculated as the total pen feed intake/total pen weight

^a^Lameness and seizures due to Aerococcus viridians meningitis

^b^Polyarthritis due to *Streptococcus suis*

**Table 5:** Clinical scores, lung lesions and *M. hyopneumoniae* (Mhyop) counts in the lungs in the dual challenge study in piglets. All piglets were challenged with *M. hyopneumoniae* on days 0, 1 and 3 and with PRRSV on day 14. Pigs in treated-challenged group were medicated daily with tylvalosin at 20 mg/kg body weight from day 10 to day 20. Treatement description is in Table 5

Group	***n***	Clinical scores		Lung scores mean ± SD		Mhyop isolation	
		Day 14	Day 24	Consolidation	Congestion	***N*** positive (%)	Mean colony forming units (cfu)/mL ± SD
Treated- challenged	7	0.5 ± 0.53	3.1 ± 1.86	14.7 ± 10.0	66.7 ± 24.9	0 (0%)	0
Untreated-challenged	7	1.0 ± 1.15	2.9 ± 2.19	22.1 ± 11.0	57.6 ± 21.0	All (100%)	2.68 × 10^4^ ± 2.72 × 10^4^
*P*-value		0.261	0.833	0.184	0.431	Not compared	0.023

#### Cytokine response

**Fig. 5:**
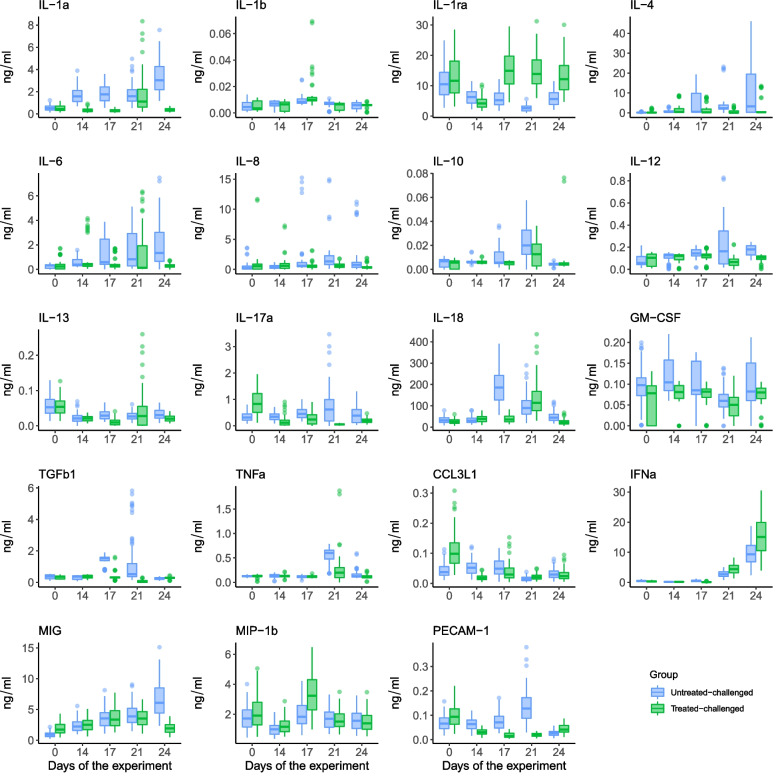
Changes in cytokine production in time from the start of the dual infection study in piglets. The box-and-whisker plots depict the minimum, first quartile, median, third quartile and the maximum temperature values observed at each day within each group. Individual points are observed outliers. Statistical differences are described in the text of the manuscript. Treatment description is in Table 7

Tylvalosin treated-challenge piglets had statistically significant lower serum levels of IL1β (*P* < 0.001), IL-4 (*P* < 0.001), IL-6 (*P* < 0.001), IL-8 (*P* < 0.001), IL-10 (*P* < 0.001), IL-12 (*P* < 0.001), IL-1α (*P* < 0.001), IL-13 (*P* < 0.001), IL-17A (*P* < 0.001), IL-18 (*P* < 0.001), GM-CSF (*P* < 0.001), TGFβ1 (*P* < 0.001), TNFα (*P* < 0.001), CCL3L1 (*P* < 0.001), MIG (*P* < 0.001), PEPCAM-1 (*P* < 0.001) when compared to the untreated-challenged piglets (Fig. 5). In contrast, treated-challenged piglets had significantly higher levels of IFNα (*P* < 0.001), IL-1ra (*P* < 0.001), MIP-1b (*P* < 0.001) when compared to untreated-challenged piglets (Fig. 5).

**Fig. 6:**
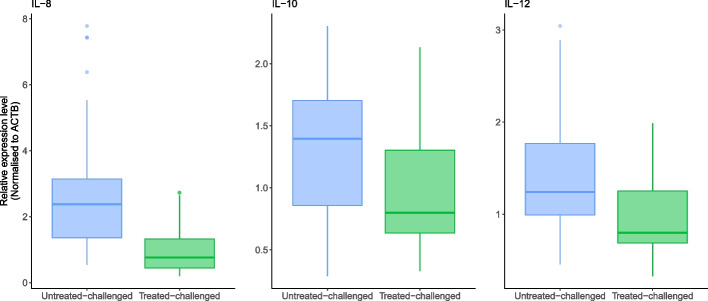
Cytokine levels in lung homogenate (day 10 post challenge) of the dual infection study in piglets. Statistical differences are described in the text of the manuscript. Treatment description is in Table 7

In lung homogenate, tylvalosin treated-challenged animals had significantly lower levels of IL-8 (*P* < 0.001), IL-10 (*P* < 0.001) and IL-12 (*P* < 0.001) when compared to control pigs (Fig. 6). Although not significant TNFα levels were also numerically lower in lung homogenates from tylvalosin treated piglets compared to untreated pigs (*P* = 0.082).

#### PRRSV in serum and lung tissue from piglets by PCR

Piglets from both groups were positive for PRRSV in serum from day 17 (3 days post challenge). Results are summarised in Table 6 and show no biologically meaningful differences between groups.

**Table 6:** PRRSv PCR log 10 virus copies in serum and bronchoalveolar lavage (BAL) in the dual challenge study in piglets. All piglets were challenged with *M. hyopneumoniae* on days 0, 1 and 3 and with PRRSV on day 14. Pigs in treated-challenged group were medicated daily with tylvalosin at 20 mg/kg body weight from day 10 to day 20. Treatement description is in Table 5

Group	Unit	PRRSv PCR in serum on different days (log 10 virus copies/ml)				PRRSv PCR in BAL from lungs (virus copies)
		Day 14	Day 17	Day 21	Day 24	
Treated- challenged	Mean	0	8.73	8.59	7.49	6.95
	SD	0	0.21	0.20	0.87	0.62
	N	8	8	8	7	7
Untreated-challenged	Mean	0	8.50	8.04	6.89	6.36
	SD	0	0.16	0.66	0.57	1.06
	N	8	7	7	7	7
	*P*-value	1.000	0.056	0.017	0.154	0.295

Virus was present in BAL from all pigs in both groups, with no significant differences noted (Table 6).

#### Bacteriology

At necropsy, statistical differences were noted in the ability to recover *Mhyop* from treated-challenged piglets compared to untreated-challenged piglets. Specifically, lung lavages from treated-challenged piglets were all negative for *Mhyop,* whereas lung lavages from untreated-challenged piglets were all positive. The *Mhyop* counts (cfu/mL lavage) were statistically lower in tylvalosin pigs compared to untreated pigs (*P* = 0.023; Table 5).

## Discussion

Previous in vitro investigations have indicated that tylvalosin might have anti-viral effects against PRRSV either by inhibiting its replication [14], or via immunomodulation [9, 18]. This study aimed to investigate the in vivo effect of administering tylvalosin in sows and piglets using either PRRSV-2 alone or dual pathogen (*Mhyop* and PRRSV-1) challenge models.

Both models were highly effective in causing infection and pathology. The PRRSV-2 challenge in sows resulted in significant losses in reproductive performance and high preweaning mortality which are typical manifestations of PRRSV infection in sows [6]. The dual challenge model in piglets effectively induced moderate PRDC, characterised by respiratory disease, lung consolidation and congestion [3, 6].

Respiratory diseases in pigs are well reported to result in significant increases in proinflammatory cytokines [5, 7, 8, 21]. During early infection, TNFα, IL-1α, IL-6 and IL-8 are produced and result in stimulation of local and systematic pro/anti-inflammatory responses [5]. In PRRSV and/or *Mhyop* infections, high levels of TNF-α, IL-1, IL-6, IL-8, IL-10 and IL-12 have been previously observed in the lungs and blood [7, 8, 21–27]. Similarly, in both studies in this manuscript, increased levels of several of these cytokines were observed after PRRSV challenge both locally (uterus and lungs) and systemically (serum).

The immunomodulatory activities of macrolides have been deliberated for some time as they could play a role in the treatment of clinical respiratory infections in animal models [11] particularly because of beneficial anti-inflammatory effects [28]. These two studies support the immunomodulatory effects of macrolides as the expression of several cytokines were affected by tylvalosin treatment.

From all the investigated cytokines, IL-8, IL-12, TNFα and GM-CSF seem especially relevant as they were consistently increased post challenge both in the PRRSV challenge study in pregnant sowsPRRSV and in the dual challenge study in piglets.

Of special interest are TNFα and IL-8 as they were expressed less in animals treated with tylvalosin, both systemically (serum) and locally (uterus or lungs). These findings are consistent across studies and agree with previous in vitro findings [18]. TNFα is a cell signalling/regulation protein which can have pro-inflammatory/apoptotic or anti-inflammatory/antiviral effects and may have a role in pulmonary defence against PRRSV [29, 30]. The role of TNFα in PRRSV pathology is somewhat contradictory given that in some models there is very little induction [5], while in others show significant expression at certain time points [7]. Expression of TNFα has been positively correlated with severity of porcine respiratory disease [5, 31]. IL-8 is a chemotactic, pro-inflammatory cytokine secreted on induction of apoptosis in bronchiolar epithelial cells. Apoptotic neutrophils have to be cleared through efferocytosis by macrophages to prevent the release of toxic neutrophil granules which if delayed results in inflammation-related tissue damage [10]. As with TNFα, aberrant production of IL-8 could lead to damage of healthy tissue [10].

IL-10 and IL-12 also were locally reduced to some extend by tylvalosin in both studies. In the dual challenge study, both were expressed less in lungs from piglets treated with tylvalosin. In the PRRSV single challenge study, compared to untreated-challenged sows, both cytokines were also reduced by tylvalosin in the fluid from the lumen of the reproductive tract by approximately 50%, although the reduction was not statistically different likely due to sample size. Upregulation of IL-10 has been shown after PRRSV infection and is associated with impaired immune response [27]. IL-12 is a T and natural killer (NK) cell stimulating factor increasing production of TNFα and IFN-γ and increasing cytotoxic activity. It has been reported to be upregulated in pulmonary alveolar macrophages and bronchial lavage fluid in pigs infected with PRRSV and/or *Mhyop* [7].

In contrast, tylvalosin appeared to up-regulate levels of some cytokines in the dual challenge piglet model that are thought to play a role in damping down the immune response. In serum, increased levels of IFN-α, IL-1ra and MIP-1β(CCL4) were observed post PRRSV challenge. Upregulation of IL-1ra could help explain the statistically lower levels of TNFα as it can modulate the production of both TNFα and IL-1 [32]. Pigs have been shown to generate a poor IFN-α response following PRRSV infection, increasing this response may lead to improved PRRSV outcomes as when provided in a nonreplicating human adenovirus type 5 vector (Ad5-pIFN-α) lower viremia was observed [33].

Less inflammation by immunomodulation of all of these cytokines when tylvalosin was administered, may have therefore reduced the overall severity of disease. Indeed, Zhao et al. (2014) noted improved growth performance and reduced lung pathology in piglets treated with tylvalosin and Toda et al.*,* also noted improved growth and reduced mortality in nursery pigs receiving tylvalosin as an in-feed premix [19, 20]. Similarly, in the dual challenge study, piglets in the tylvalosin group tended to have less severe disease and better performance.

Studies have shown that PRRSV reproductive pathology could be the result of damage to maternal tissues, or production of maternal factors that negatively affect the foetus [34]. Endometritis and myometritis are often found in PRRSV infected sows in association with umbilical cord lesions in the foetuses. These lesions could result in foetus hypoxia and reproductive failure [35]*.* In the study in sows, the effect of tylvalosin on reproductive performance in this sow study was limited. Whether tylvalosin resulted in less inflammation with a positive impact on reproduction is unknown. The data on piglet survival to weaning is somehow inconclusive due to limited sample size. However, simulated numbers for expected weaned production were generated to evaluate the relevance of these findings in a commercial production system. The simulations were based on the sow study data and analysis results and indicate that per 1000 sows 122 pigs more would be weaned on average in sows that were treated with Aivlosin (data not shown). Recent work has shown that a difference of 24.52 piglet per 1000 sows is clinically relevant in PRRSV affected herds as it is the difference observed between stable and non-stable farms [36]. Therefore, the difference observed in piglet survival in our study could be clinically relevant.

The anti-viral activity reported for tylvalosin in vitro was not replicated in vivo in the dual challenge study in piglets at the dose levels used. Results from serum samples showed that the viral load measured by RT-qPCR was slightly higher for tylvalosin-treated pigs than for the controls on certain days. However, measurements in both groups were within the same log_10_ range and the finding is probably of no biological relevance, especially considering that there were no differences in viral loads in the lungs. Results of RT-qPCR should be interpreted with care as they do not distinguish active from inactive PRRSV replication, and the authors can only speculate regarding this finding. Considering that tylvalosin reduced Mhyop loads in all treated pigs, it is possible that the lower presence of of *Mhyop* in the lungs would have favoured PRRSV replication as the primary pathogen, resulting in more infected cells and consequent higher viremia. As an example, a previous report of a dual Mhyop-PRRSV study, showed more cells infected by PRRSV in lungs from pigs challenged with PRRSV alone compared to pigs first challenged with *Mhyop* and then with PRRSV 21 days later [37]. It is noted that treatment with the macrolide tilmicosin did not seem to reduce viremia in another study, in fact, pigs treated with tilmicosin also tended to have higher viral RNA copies in serum than untreated pigs [38].

However, in the sows study some virological observations did favour the tylvalosin treated group. For example, in that study no piglets born from tylvalosin treated sows were positive in faecal swabs at weaning. In our sow study, less sows seemed to be shedding virus in faeces on day 21 (around parturition) and it is possible that piglets from these sows had less exposure to the virus as a consequence. It is also possible that this trend in reduced viremia in piglets was associated with the fact that no piglets from tylvalosin sows had lung lesions at weaning and more piglets survived to weaning. More presence of PRRSV in foetus tissue has for example been associated with foetal pathological lesions that would result either in reproductive failure or weaker piglets [39].

In addition to the immunomodulatory effects, the piglet dual challenge study confirmed that tylvalosin, at the high target experimental dose rate used (20 mg/kg for 10 days), was highly efficacious in reducing the loads of *Mhyop* in the lung, with apparent elimination of the pathogen from all pigs. Macrolides are usually considered to be bacteriostatic, but at licensed doses lower than those used in these studies, tylvalosin has been found to clean the infection from lungs of pigs challenged with *Mhyop* [17]. The results of this study therefore support mycoplasmicidal effect of tylvalosin when used according to label.

## Conclusion

Tylvalosin reduced *Mhyop* counts in the lungs and showed a highly significant immunomodulatory effect on multiple cytokines in both sows and piglets challenged with *Mhyop* and or PRRSV. However, there was no evidence of anti-viral activity against PRRSV as determined by viral counts. These findings indicate that tylvalosin if used judiciously in operations with coexisting *Mhyop* and PRRSV infections, will have a measurable impact on animal health. These studies also confirm the local regulatory effects on inflammatory mediators implied by the present data. Further investigation is needed to characterise the anti-inflammatory benefits of tylvalosin at clinically relevant dose rates in larger group numbers.

## Materials and methods

Both studies were conducted in accordance with the principles of Good Clinical Practice (GCP) and followed a randomised, controlled, masked and parallel study design [40]. Persons involved in the clinical assessments, lung scoring, bacteriological, virological and cytokine assessments were maintained unaware of the treatment allocation throughout, except for the untreated-unchallenged control in the sow study. Personnel involved in administering the medications were unmasked and did not participate in any efficacy assessments. A summary of the treatment description in each study can be found in Table 7.

**Table 7:** Treatment groups

			Challenge		Water treatment	
Study	Group	***N***	***Mhyop***	PRRSV	Treatment	Dose (mg/kg/day)
PRRSV in sows	Untreated-Unchallenged	6	None	None	None	–
	Untreated-Challenged	6		85–89 Days Gestation^a^	None	–
	Treated-Challenged	6			Tylvalosin -3DPC to farrow	5
Dual challenge piglets	Untreated-challenged	8	Days 0,1 and 2	Day 14	None	–
	Treated-challenged	8			Tylvalosin Day 10 to 19	20

^a^ as determined by last recorded insemination day

*Mhyop Mycoplasma hyopneumoniae*, *PRRSV* Porcine Reproductive and Respiratory Syndrome Virus

### PRRSV challenge study in pregnant sows

#### Animal husbandry and housing

Eighteen healthy, commercial pregnant sows from parity four which were seronegative and real-time reverse-transcriptase quantitative (RT)-q polymerase chain reaction (PCR) negative for PRRSV Types 1 and 2 and real-time PCR negative for porcine circovirus type 2 (PCV2), *Mhyop*, and porcine parvovirus (PPV) were used in this study. Sows also had titres of < 800, as determined by microscopic agglutination test (MAT) against six serovars of *Leptospirosis* (canicola, ictero, grippo, pomona, hardjo, and bratislava). All laboratory analysis was undertaken at Iowa State University using accredited tests.

For randomisation, sows were blocked by the number of piglets born in the sow’s previous parity. Each block consisted of 3 sows and sows within each block were randomly assigned to either untreated-unchallenged (Negative control), untreated-challenged (positive control) or treated-challenged (tylvalosin) (Table 4). Sows were housed within individual farrowing crates, containing mats and heat lamps and were acclimatised for seven days prior to the start of the study. The sows were housed in BSL-2 facilities. The challenged sows, both treated and untreated, were housed in the same room, whereas untreated unchallenged sows were housed in a separate room to avoid cross contamination. The accommodations had artificial lighting and ventilation and was maintained at an appropriate temperature. Sows were fed a commercially available antibiotic-free feed once daily. For all groups, water was provided ad libitum through drinking nipples.

#### Treatments

Sows in the tylvalosin group received a water medicated with Aivlosin® 625 mg/g granules for use in drinking water from 3 days prior to PRRSV challenge until the first sow in the group farrowed. The average daily dose calculated based on nominal inclusion rate in water and measured water intake was 5 mg tylvalosin/kg body weight/day. Medicated water was provided with a proportioner system (Dosatron) connected to the water line. Two sows refused to drink the medicated water from the nipples and therefore received their dose via a top dressing with concentrated water on their feed. These two sows were then provided fresh drinking water in their feed mangers. The untreated-unchallenged and untreated-challenged sows received unmedicated water all through the study. To maintain masking, the water line of the control group was connected to a proportioner system. Piglets were offered a creep feed starting at 10 days of age. All pigs were euthanised by injection of a cocktail of anaesthetics followed by exsanguination either on scheduled necropsy day or when withdrawn from the study.

#### PRRSV challenge

Each sow was challenged intranasally on day 0 (85–89 days of gestation) with 2 mL per nostril (4 mL total) of North American PRRSV 1–7-4 at 1 × 10^6^ log_10_ 50% tissue culture infectious doses per mL (TCID_50_) per animal using a syringe fitted with an atomizing tip. Challenge was dispensed in one nostril at a time gradually upon inhalation and sow remained snared with snout elevated to insure challenge inhalation. The challenge material was free from *Mhyop*, *M hyosynoviae*, *M hyorhinis*, porcine rotavirus A, B, C, porcine epidemic diarrhoea virus, transmissible gastroenteritis virus, porcine deltacoronavirus, porcine parvovirus, porcine teschovirus, porcine sapelovirus, swine Influenza A virus and porcine circovirus 2 and 3, as determined by real-time PCR. All laboratory analysis was undertaken at Iowa State University using accredited tests with the exception of determining the concentration of the challenge virus (TCID_50_) which was performed at RTI (Research | Technology | Innovation).

#### Sampling and analyses

Sow blood, oral and faecal swabs were collected on − 3, 0, 14, 21 days post challenge and on 0, 2, 9, 16, 21–25 post farrow. Piglet blood, oral and faecal swab samples and weight were collected on 0, 2, 9, 16, day of planned necropsy (24–28 days) post farrow or on the day the piglet was euthanised or found dead. At post-mortem, a range of tissues were collected from both sows (thymus, lung, iliac lymph node [ILN], mesenteric lymph node [MLN], tonsil, reproductive tract luminal fluid) and piglets (thymus, lung, tonsil, spleen). Blood, oral and faecal swabs along with fresh tissues were used for real-time RT-qPCR at Iowa State University. Blood samples were also used to determine the serum cytokine response to 20 targets [Interleukins: IL1β, IL-4, IL-6, IL-8, IL-10, IL-12, IL-1α, IL-1 receptor antagonist [ra], IL-13, IL-17A, IL-18, Granulocyte-macrophage colony-stimulating factor [GM-CSF], Interferons: IFNγ, IFNα, transforming growth factor [TGF]β1, tumour necrosis factor [TNF] α, Chemokines: CCL3L1, Monokine induced by gamma [MIG], Macrophage Inflammatory Protein [MIP]-1b, PEPCAM-1) (Ray Biotech). The same cytokines were analysed in reproductive tract luminal fluid. The cytokine analysis was performed at RTI with analysis undertaken at Ray Biotech.

#### Clinical and pathological observations

Sow temperatures, nasal discharge, coughing, demeanour and respiratory rate were recorded daily from day 0 to day 20 post challenge.

Individual sow water intake was recorded for the untreated-challenged and treated-challenged groups, mean water intake was recorded for the untreated-unchallenged control group.

Sows farrowed between 113 and 119 days gestation except for one sow (84210) who was euthanized on day 17 post challenge due to gastric ulcer. Piglet viability was recorded at the time of farrowing and were categorised into live born viable (LBV), live born non-viable (LBNV-born live but had failure to thrive or were non-ambulatory), Stillborn (SB - piglets born dead, but which were not mummified), or mummified (MUM). Sow and piglet (surviving to weaning) lungs were evaluated and the percentage of area of each individual lobe affected by pneumoniae (0 to 100%) was recorded.

### Piglet dual challenge study

#### Animal husbandry and housing

A mixed sex group of 18 piglets 3–4 weeks of age from Large White/Landrace sows crossed with a Duroc boar. The piglets originated from a high health status farm, with known history of being free from *Mhyop* disease. The piglets were confirmed negative for antibodies to *Mhyop* and PRRSV, as well as PCV2 by ELISA prior to first challenge. They were blocked by weight and randomly allocated to two separate pens in a shared airspace within environmentally controlled biosecurity housing. Solid separation between pens did not allow nose-to-nose contact. Each pen was then randomized to either treated-challenged (tylvalosin) or untreated-challenged (positive control) (Table 4). The bedding was a deep litter straw system with fresh straw added as required. A commercial antibiotic free pig meal was fed ad libitum all through the study. Water to each pen was supplied from separate reservoir drinkers to allow for the provision of medication. After an acclimatization period (7 days), the 8 heaviest piglets from each group were selected for inclusion in the study. Bodyweights were recorded on days 0, 14 and 24.

#### PRRSV and *Mhyop* challenge

All pigs were infected with *Mhyop* on days 0, 1 and 2; and with PRRSV on day 14. For both pathogens, inoculation was via the intranasal route with 2.5 mL of each inoculum being administered into each nostril using a syringe fitted with an atomizing tip.

Each piglet was challenged intranasally using a syringe and an aerosol adapter on day 0, 1 and 2 with 2.5 mL per nostril (5 mL total) of *Mhyop* strain 42P11 at 1.95, 2.25 and 1.58 × 10^8^ cfu/mL respectively. The minimum inhibitory concentration (MIC) for this *Mhyop* strain had been previously determined in vitro to be 0.015 μg tylvalosin/ml [17].

Each piglet was challenged intranasally on day 14 with 2.5 mL per nostril (5 mL total) of PRRSV-1, subtype 2 (LT − 3) [41] at 1.56 × 10^6^ TCID_50_/mL.

#### Treatments

The commercial formulation containing tylvalosin tartrate (Aivlosin® 625 mg/g granules for use in drinking water, ECO Animal Health) was administered to pigs in the tylvalosin group daily from day 10 to day 20 post *Mhyop* challenge, i.e. 4 days prior to PRRSV challenge until 6 days post PRRSV challenge. The average dose of tylvalosin from day 10 to 20 was 22.59 mg/kg based on measured water intake and inclusion rate of tylvalosin in water. Pigs in the control group received equivalent volumes of water without medication. Drinking water was prepared on the basis of the body weight of the heaviest pig to provide a target dose of at least 20 mg tylvalosin/kg body weight/day for 10 consecutive days. The volume of water supplied was based on estimated water consumption from water intake measurements starting on day 8.

#### Sampling and analyses

Blood samples were collected on days 0, 14, 17, 21 and 24 and lung lavage/homogenates were taken at post-mortem. All pigs were euthanised by lethal injection of pentobarbital sodium on day 24, the lungs were removed at necropsy, scored, trimmed, weighed and samples collected. After each lung had been scored, the lungs were divided into left and right at the tracheal bifurcation. One lung was then lavaged using 50 mL of phosphate buffered saline, introduced into the bronchi via a funnel. The lung was then massaged and the fluid recovered by inverting the lung over a sterile container. Lung lavage samples for bacteriology were processed shortly after collection. For *Mhyop* counts, lung lavage was placed in Mycoplasma broth and thereafter inoculated onto Mycoplasma agar plates (Mycoplasma Experience). The resultant colonies were counted at Moredun (Midlothian, UK) after incubation for 10 to 14 days.

Serum samples and lung homogenates/lavages were used to measure PRRSV viral load using real-time RT-qPCR [42] and also to quantify cytokine concentrations for 20 [Interleukins: IL1β, IL-4, IL-6, IL-8, IL-10, IL-12, GM-CSF, IFN-γ, TGFβ1, TNF-α, chemokine ligand 3-like 1 (CCL3L1), IFN-α, IL-1α, IL-1ra, IL-13, IL-17A, IL-18, MIG/CXCL9, MIP-1β/CCL4 and PECAM-1/CD31 (Abcam plc, Cambridge). Viral load analyses by RT-qPCR were performed at the Animal and Plant Health Agency (APHA, Surrey, UK) and cytokines analyses were done at The Roslin Institute (Midlothian, UK).

#### Clinical and pathological observations

Clinical examinations were performed twice daily from day 14 to day 24, with rectal temperatures, nasal discharge, coughing, demeanour and respiratory rate and effort being recorded and scored in accordance with Table 8. A composite total score (sum of each individual score) was used for comparison on day 14 and day 24.

**Table 8:** Clinical parameters and scoring system in the dual challenge (PRRS+Mhyop) study in piglets

Parameter	Classification and score			
	Normal Score = 0	Mild Score = 1	Moderate Score = 2	Severe Score = 3
Rectal temperature	37.5–39.5 °C	39.6–40.0 °C	40.1–40.9 °C	≥41.0 or < 37.5 °C
Demeanour	Normal	Reduced activity, reduced appetite	Reluctance to rise, reduced appetite	Recumbent, moribund
Nasal discharge	Absent	Serous	Seromucoid	Mucoid
Coughing	Absent	1–2 dry coughs	> 2 dry coughs or 1–2 productive coughs	> 2 productive coughs
Respiration	Normal	Slightly increased respiratory rate or effort	More pronounced increased respiratory rate and effort	Respiratory distress, open mouth breathing

Lungs were scored by visual examination and palpation, to determine the percentages of gross pathology present in each of the lung lobes, differentiating between consolidation and congestion where possible. The percentages were then weighted based on the ratio of each lobe to total lung mass as follows: left apical 6%, left cardiac 10%, left diaphragmatic 31%, right apical 5%, right cardiac 10%, right diaphragmatic 30%, and intermediate 8%. The weighted lung lobe values were then summed for each animal to yield the percentage of total lung lesions both for consolidation and congestion.

#### Statistical methods

*PRRSV challenge study in pregnant sows* Primary (response) variables for analysis were PRRSV viral loads of positive samples in serum, faecal and oral swabs and tissues, piglet viability at farrowing and piglet survival and body weight at weaning (survival). Other variables assessed were clinical observations of the sows until day 21 post challenge, cytokine dynamics and lung lesions in sows and piglets at weaning.

For analysis, linear regression models with or without random effects to account for clustering (e.g., Piglets from the same litter) and/or repeated measures (multiple samples taken in time from the same animal) were used. In these models, the explanatory variables were the experimental group (for between group comparisons), their potential interactions with time variables (e.g., day of the experiment or age of piglets) and when relevant sample type (e.g., models assessing shedding). To deal with non-linear relationships between the response variable and the time variables, cubic splines were used when these variables were included in the models. Model selection was based on the Akaike Information Criterion (AIC). The models’ variable significance was assessed using ANOVA test. Biomarkers in reproductive tract lumen were analysed with t-test or Wilcoxon-Mann-Whitney test if the biomarkers met or did not meet normality criteria across the groups, respectively.

To assess piglet viability at farrowing the proportion of piglets born alive (and viable) were assessed using generalised mixed logistic regression models with random effects to account for clustering effects or repeated measures. To assess survival at weaning, a Cox proportional hazards models were used which included random effects to account for clustering effects or repeated measures. In these models, group was used as explanatory variable for between group comparisons. Simulated data of expected number of weaned piglets were generated based on the experiment data and analysis results. In these simulations the following sources of variation (per group) are included: 1) Number of piglets a sow farrows, 2) Probability piglets are born alive and 3) Probability piglets survive at weaning. Simulations were done using R.

The threshold for significance was set to *p* < 0.05. A Bonferroni correction was applied to adjust *p* values when multiple comparisons were performed. All the analysis was performed using the statistical software R version 4.0.2 [43]. The library “lme4” [44] and “splines” [43] were used for fitting the generalised or liner regression models with random effects and the libraries “survival” [45] and “coxme” [46] were used for the survival analysis.

##### Piglet dual challenge study

Cytokines in serum and lung were analysed fitting linear regression models with or without random effects to account for repeated measures on the same piglet. In these models, the explanatory variables were the experimental group (for between group comparisons), day of the experiment and the potential interactions between group and time. To deal with non-linear relationships between the response variable and the time variables, cubic splines were used. Model selection was based on the Akaike Information Criterion (AIC). The models’ variable significance was assessed using ANOVA test.

## Data Availability

Due to confidentiality agreements with research collaborators and commercially sensitive nature of the research, data are subject to access restriction. Please contact the corresponding author for any request.
